# Claudins in Renal Physiology and Pathology

**DOI:** 10.3390/genes11030290

**Published:** 2020-03-10

**Authors:** Caroline Prot-Bertoye, Pascal Houillier

**Affiliations:** 1 Centre de Recherche des Cordeliers, INSERM, Sorbonne Université, Université de Paris, F-75006 Paris, France; caroline.bertoye@aphp.fr; 2 Service de Physiologie, Hôpital Européen Georges Pompidou, Assistance Publique-Hôpitaux de Paris, F-75015 Paris, France; 3 Centre de Référence des Maladies Rénales Héréditaires de l’Enfant et de l’Adulte (MARHEA), F-75015 Paris, France; 4 Centre de Référence des Maladies Rares du Calcium et du Phosphate, F-75015 Paris, France; 5 CNRS, ERL8228, F-75006 Paris, France

**Keywords:** claudin 10b, claudin 16, claudin 19, claudin 14, kidney, tight junction, HELIX syndrome, familial hypomagnesemia with hypercalciuria and nephrocalcinosis, sodium, divalent cations

## Abstract

Claudins are integral proteins expressed at the tight junctions of epithelial and endothelial cells. In the mammalian kidney, every tubular segment express a specific set of claudins that give to that segment unique properties regarding permeability and selectivity of the paracellular pathway. So far, 3 claudins (10b, 16 and 19) have been causally traced to rare human syndromes: variants of *CLDN10b* cause HELIX syndrome and variants of *CLDN16* or *CLDN19* cause familial hypomagnesemia with hypercalciuria and nephrocalcinosis. The review summarizes our current knowledge on the physiology of mammalian tight junctions and paracellular ion transport, as well as on the role of the 3 above-mentioned claudins in health and disease. Claudin 14, although not having been causally linked to any rare renal disease, is also considered, because available evidence suggests that it may interact with claudin 16. Some single-nucleotide polymorphisms of *CLDN14* are associated with urinary calcium excretion and/or kidney stones. For each claudin considered, the pattern of expression, the function and the human syndrome caused by pathogenic variants are described.

## 1. Introduction

Almost every epithelium in multicellular organisms separates two compartments (apical and basolateral) with differing ion composition. Depending on the nature of the compartments, human epithelia can behave as barriers (e.g., the skin epithelium) or allow selective transport of solutes and/or solvent from apical to basolateral compartment or vice-versa (e.g., intestinal or renal tubular epithelia). The asymmetry of composition between apical and basolateral compartment is created and maintained by active transport (with the notable exception of passive transport that occurs as a result of Gibbs-Donnan effect). Active transport can occur transcellularly only, at the expense of energy release; for example hydrolysis of ATP releases energy used to actively pump ions across the plasma membrane and to build transmembrane electrochemical gradients. Passive diffusion between 2 compartments may be defined as the flow of solute that occurs in response to the difference in electrochemical potentials of the considered solute between both compartments. In an epithelium, diffusion can occur across plasma membranes or along the paracellular pathway. Because of the existing transmembrane electrochemical potential differences, sodium (Na^+^), calcium (Ca^2+^) and magnesium (Mg^2+^) cannot cross passively both apical and basolateral membranes: therefore, at some step, transcellular transport of Na^+^, Ca^2+^ and Mg^2+^ requires energy expenditure. By contrast, passive paracellular diffusion of Na^+^, Ca^2+^ and Mg^2+^ allows both transepithelial transport and energy saving [1]. The present review is a summary of our knowledge of normal and abnormal paracellular ion transport in the mammalian renal tubule. Specifically, it focuses on the role of claudin proteins, which are highly specialized proteins expressed at the tight junction, in health and disease.

## 2. Structure and Function of Tight Junction in the Kidney

The properties of the paracellular pathway along the renal tubular epithelium have been less intensively studied than the properties of the transcellular pathway. Nevertheless, epithelia have been classified as leaky or tight, according to the value of transepithelial resistance, R_T_. Because transcellular and paracellular pathways are organized in parallel, the reciprocal of transepithelial resistance equals the sum of the reciprocal of transcellular resistance (R_C_) and of the reciprocal of paracellular resistance (R_P_), according to Ohm’s law,

$$\frac{1}{R_{T}}=\frac{1}{R_{C}}+\frac{1\;}{R_{P}}.$$

The direct consequence, assuming that R_C_ is high and almost constant, is that «leaky» epithelia (low R_T_) must have low R_P_ and «tight» epithelia (high R_T_) have high R_P_. Several investigators have measured R_T_ in the various segments of the renal tubule of rodents and rabbits; they consistently found that the proximal tubule has the lowest R_T_ of all segments and that R_T_ increases to a maximal value in the inner medullary collecting duct (for review, see Reference [2]). Besides electrical resistance, every paracellular pathway is also characterized by its selectivity to ion charge and size [3,4,5]. The size selectivity of paracellular pathways was studied by several investigators, mostly in cells lines or intestinal epithelia. Most studies reported that at least two populations of pores are present in the paracellular pathway, the most restrictive having a pore size of 4–8 Å [6,7,8,9,10]. The diameter of the major cations in extracellular fluid is 6.62–8.60 Å when they are in their hydrated form and 1.44–2.98 Å when they are in their unhydrated form [11]. The selectivity to ion charge vary from epithelium to epithelium: some are cation selective whereas other are anion selective. Most of the resistance and charge selectivity of the paracellular pathway are determined at the tight junction. Tight junction is formed by a complex of multiple proteins, subdivided into transmembrane proteins and cytoplasmic plaque proteins, including signaling proteins and scaffolding proteins (adapters that link the tight junction complex to the actin cytoskeleton) [12,13]; tight junction contacts and their selective permeation properties are created by a large family of transmembrane proteins called claudins [14,15,16]. In mammals, the claudin gene family is composed of at least 27 members [2,17,18]. Claudins are relatively short transmembrane proteins that possess between 207 and 305 amino acids. The calculated molecular mass is between 21 and 34 kDa. The general structure of all claudins is similar, consisting in a short intracellular NH_2_ terminus, a longer intracellular COOH terminus, two extracellular segments (ECS1 and ECS2) (which form a β-sheet of five β-strands (4 β-strands in ECS1 and 1 β-strand in ECS2)), one intracellular loop and four transmembrane domains (helices). The first extracellular segment ECS1 contains the common motif W-LW-C-C and positively and negatively charged amino acid residues that determine the charge selectivity. This motif is critical for folding ECS1 in a region close to the plasma membrane and to form the characteristic β-sheet fold of ECS1 and ECS2 [19]. Claudins polymerize in tight junctions strands through cis (between two claudin within the same cell membrane) and trans (between claudins on opposing cell membranes) interactions [17] (Cis-polymerization would involve a short extracellular helix (ECH) at the end of ECS1 and the extracellular extension of the third transmembrane domain TM3 of a neighboring claudin [17]. Trans-interaction of claudins between neighboring cells is presumed to involve the intercellular interaction of the variable regions V1 (in ECS1) and V2 (in ECS2) [17,20,21]. An hypothetical model has been proposed: tight junction strands would consist of anti-parallel double-rows of claudins and the paracellular channel would be achieved by the interaction of the four claudins coming from double-rows [22]. A trans-interacting octamer model with a double pore has also been proposed [21,23]).

Among the claudin family, a highly conserved PDZ -binding domain is present near the end of the carboxy-terminal end, which binds to the homologous domain of scaffolding proteins such as ZO-1, which is important for the localization of claudins at the tight junction [24,25]. The COOH terminus also contains a number of potential phosphorylation sites, reported to play a role in the localization of claudins at the tight junction.

**Figure 1:**
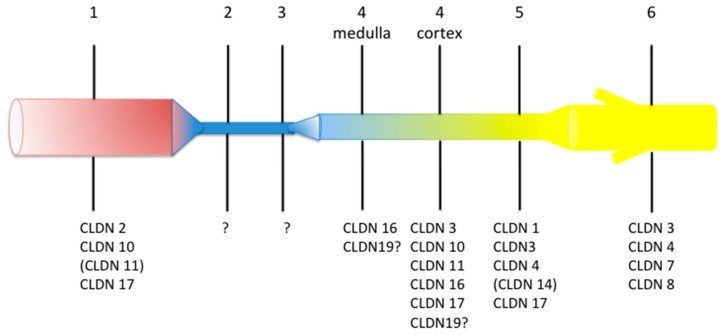
Pattern of expression of claudin (CLDN) proteins along the human renal tubule and collecting duct. The figure is a summary of data published in References [26,27,28,29] (the expression pattern in rodent kidney has recently been summarized in several reviews [2,30,31]). Question mark means that direct evidence of the expression of the considered claudin in human is not available but that indirect evidence suggests that it should be expressed there. Brackets mean that the expression is low. The numbers above the various segments are: 1, proximal convoluted and straight tubule; 2, descending thin limb; 3, ascending thin limb; 4, distal straight tubule (thick ascending limb); 5, distal convoluted tubule; 6, cortical and outer medullary collecting duct. Colors illustrate the fact that composition and/or volume of tubular fluid changes along the renal tubule. Readers interested in mRNA expression pattern can visit https://hpcwebapps.cit.nih.gov/ESBL/Database/NephronRNAseq/All_transcripts.html.

Many claudins are expressed along the mammalian renal tubule and collecting duct. Although some controversies remain regarding the exact localization of a specific claudin, the overall pattern is that every tubular segment expresses a specific association of claudins, likely accounting for the specific properties of the paracellular pathway of every nephron segment (Figure 1).

So far, only a few claudins have been causally involved in human monogenic diseases; they are claudin (CLDN) 10b, CLDN16 and CLDN19 and will be considered below.

## 3. Claudin 10b and the HELIX Syndrome

The human *CLDN10* gene is located on chromosome 13q31-q34 and contains five exons [32]. There are two claudin 10 splice variants differing in the first exon that encode two main claudin 10 isoforms, claudin 10a and claudin 10b [33].

### 3.1. Phenotype

In 2017, three independent groups described the clinical consequences of biallelic mutations of the *CLDN10* gene.

The first report described the phenotype of two unrelated patients bearing compound heterozygous mutations (Table 1, [34]). One adult female patient had hypokalemic metabolic alkalosis, chronic kidney disease (CKD) stage 3, polyuria, low urinary Mg^2+^ excretion and a tendency toward hypermagnesemia; the second patient displayed similar abnormalities associating hypokalemic metabolic alkalosis and a tendency toward hypermagnesemia. The second report described the phenotype of 13 affected patients from 2 distantly related families, all carrying the homozygous missense mutation in exon 1b (Table 1, [35]). All affected patients had anhidrosis, heat intolerance, inability to produce tears (alacrimia) and xerostomia. A biological assessment was available in 6 among the 13 patients, aged 23–47 years. All had normal plasma potassium concentration and plasma Mg^2+^ level was either high or in the upper range of normal values. Estimated glomerular filtration rate (eGFR) was higher than 60 mL/min/1.73 m^2^. Plasma renin and aldosterone concentrations were not available. The third report described the phenotype of 6 patients from 2 unrelated families [28]. All patients were bearing one homozygous missense mutation of the *CLDN10* gene. All patients had hypohidrosis, renal loss of NaCl with secondary hyperaldosteronism and hypokalemia, as well as hypolacrimia, ichthyosis, xerostomia and severe enamel wear. Biological assessment showed plasma Mg^2+^ levels either high or at the upper limit of reference range and a blunted natriuretic and chloruretic response to furosemide infusion. The watery component of saliva was severely reduced in patients. Heterozygous family members were asymptomatic. Finally, the most recent report to date describes a teenager carrying a biallelic mutation of *CLDN10* [36]. The patient had anhidrosis, xerostomia, alacrimia and hypokalemia; he developed hypermagnesemia and CKD over a 4 years follow-up period. The acronym HELIX (Hypohidrosis, Electrolyte disturbances, hypoLacrimia, Ichthyosis, Xerostomia) has been coined to name this syndrome (Online Mendelian Inheritance in Man (OMIM) #617671).

The similarities and differences between the various reports are summarized in Table 1. All patients with the HELIX syndrome have a functional defect of sweat, salivary and lacrimal glands; most of them have blood electrolyte abnormalities, hypermagnesemia and/or hypokalemia; 25% have CKD stage 3 and none has nephrocalcinosis.

### 3.2. Variants/Pathogenesis

Seven pathogenic, or likely to be pathogenic, nucleotide abnormalities have been reported in patients with HELIX syndrome, so far (Table 2). Three of them are located into exon 1b and affect exclusively the protein CLDN10b; 4 are into exon 2, 3 or 4 and affect both CLDN10a and CLDN10b protein. However, no obvious difference can be seen between the phenotypes of patients bearing only mutated CLDN10b and patients carrying mutated CLDN10a and CLDN10b, which does not help to understand the specific role of the former.

The regular pattern of expression of claudin 10 isoforms in the mammalian kidney is a matter of controversy. In in situ hybridization experiments, Van Itallie and coworkers showed that *Cldn10a* transcripts were predominantly expressed by cortical tubules, whereas *Cldn10b* transcripts were predominantly expressed in tubular segments located in the outer medulla [33]. A more detailed pattern was reported by Günzel and collaborators [38]—they reported that *Cldn10a* transcripts were expressed in the proximal convoluted tubule, cortical thick ascending limb (C-TAL) and cortical collecting duct, contrasting with *Cldn10b* transcripts tubular expression that was restricted to the medullary thick ascending limb (M-TAL) and inner and outer medullary collecting duct. Hadj-Rabia et al. and Breiderhof et al. reported partially different results—*Cldn10a* transcripts were exclusively found in convoluted and straight proximal tubule and *Cldn10b* transcripts in the M- and C-TAL; none was significantly detectable in more distal segments [28,39].

No antibody is available that helps to distinguish between claudin 10a and claudin 10b proteins. Van Itallie et al. reported that Cldn10 was strongly expressed at the tight junction of M- and C-TAL, thin ascending limb and medullary collecting duct and, to a lesser extent, at the tight junction of proximal tubule and cortical collecting duct [33].

Both in vitro (Table 3) and in vivo experiments have been conducted to unravel the function of Cldn 10b.

When heterologously expressed in MDCK-C7 or LLC-PK1 cells, both human and mouse claudin 10b decrease transepithelial resistance [33,38,40]; both human and mouse claudin 10b increase permeability (P) ratios P_Na_/P_Cl_, P_Mg_/P_Cl_ and P_Ca_/P_Cl_ in MDCK-C7 cells [38]. By contrast, mouse Cldn10b affects neither transepithelial resistance nor P_Na_/P_Cl_, when expressed in MDCK-II cells that form a low resistance epithelial layer [33].

Inconsistent results on permeability to Ca^2+^, Mg^2+^, Na^+^ and chloride may result from the endogenous expression of distinct claudins in the cell lines used in experiments. Overexpression of any claudin in a cell line with a claudin background different from that normally co-expressed with the claudin under investigation can induce variable/conflicting interaction and results [42].

Mice (10 weeks old) with a deletion of Cldn10b in the TAL (*Cldn10b* cKO) show marked hypermagnesemia with low fractional excretion of Mg^2+^ in urine, mild hyperphosphatemia, mild polyuria with inability to concentrate urine upon drinking water deprivation, elevated fractional excretion of potassium and medullary nephrocalcinosis without overt impairment in GFR [39]. Fractional excretion of Na^+^ was not consistently elevated in *Cldn10b* cKO mice. Noteworthy, most of the in vivo effects of *Cldn10b* deletion, except polyuria and high fractional excretion of potassium, were corrected by simultaneous deletion of *Cldn16* (see FHHNC, below) [43].

Several of the mutant proteins have been heterologously expressed in various cell lines. In HEK293 and MDCK-C7 cells, cell surface expression of p.P149R and p.D73N CLDN10b was not decreased, as compared to wild-type protein, whereas that of p.E157_T192del CLDN10b, p.N48K CLDN10b or CLDN10b ∆4 (exon 4 was deleted) was reduced [34,35]. When expressed in MKTAL cells [44], both p.M1? and p.S131L mutated CLDN10b proteins were weakly expressed at cell surface, as compared with wild-type protein [28].

Isolated perfused TALs from *Cldn10b* cKO mice had a higher transepithelial resistance and a lower paracellular permeability ratio P_Na_/P_Cl_ than TALs from normal littermates [39]. Based on the results described above, a consensual agreement is that the specific effect of claudin 10b is to increase the paracellular permeability to Na^+^. Actually, a lower paracellular P_Na_ in TAL, sweat glands and salivary glands may account for most of the clinical consequences of *CLDN10b* loss-of-function mutations. In the M-TAL, fifty per cent of Na^+^ is passively reabsorbed along the paracellular pathway: a decrease in P_Na_ impairs Na^+^ reabsorption, causing a renal loss of Na^+^, extracellular fluid depletion with secondary hyperaldosteronism and a renal loss of potassium; the high transepithelial voltage would elevate passive paracellular Mg^2+^ and Ca^2+^ reabsorption in the TAL. In salivary and sweat ducts, where chloride is secreted transcellularly, Na^+^ is passively secreted along the paracellular pathway. A loss-of-function mutation of *CLDN10b* would decrease Na^+^ secretion and the formation of saliva and sweat.

### 3.3. Prognosis and Treatment

The long-term prognosis is unknown; however, 25% of patients had CKD stage 3 at the time of presentation and close follow-up is advised.

No specific treatment is available to improve the condition of patients with HELIX syndrome. High NaCl and fluid intake is advised, with potassium supplements and drugs blocking aldosterone secretion and/or action when hypokalemia is present. Artificial tears and saliva can be used to relieve symptoms of eye and mouth dryness, respectively. Prolonged intense physical activity should be discouraged, particularly when the outside temperature is high, to prevent the risk of hyperthermia.

## 4. Claudin 16, Claudin 19 and Familial Hypomagnesemia with Hypercalciuria and Nephrocalcinosis (FHHNC)

Familial hypomagnesemia with hypercalciuria and nephrocalcinosis is an autosomal recessive disorder caused by variants of the *CLDN16* (OMIM #248250) and *CLDN19* (OMIM #248190) genes.

The human *CLDN16* gene is located on chromosome 3q27 and contains five exons. Two potential start codons produce either a long (305 amino acids, 33 kDa) or short (235 amino acids, 27 kDa) CLDN16 protein isoform [45,46]. It is unknown which one of the two versions (long or short) is the physiologically relevant or whether both are functional. In vitro, Hou et al. described that the short CLDN16 is expressed at cell-cell borders whereas the long CLDN16 is targeted to endosomes and lysosomes [46]. However, in other studies, full length CLDN16 is expressed at the tight junction [47,48].

The human *CLDN19* gene, located on chromosome 1p34.2, contains also fives exons, encoding CLDN19, a protein of 224 amino acids [32].

Because of the autosomal recessive pattern of inheritance, parental consanguinity is frequent [26,49]. The exact prevalence of FHHNC is unknown but it is a very rare disorder, likely to be under-diagnosed.

### 4.1. Phenotype

FHHNC is characterized by an excessive urinary loss of Ca^2+^ and Mg^2+^, resulting in hypomagnesemia and hypercalciuria. Patients have nephrocalcinosis (a parenchymal deposition of calcium–based crystal in the renal parenchyma) and renal failure that may progress toward end stage renal disease early in life [26,45,49,50,51]. Of note, hypomagnesemia can be absent [51,52].

Age at onset ranges from 0 to more to 30 years for *CLDN16* [45,49,50,52,53,54,55,56] and *CLDN19* mutations [50,51,57,58] and the diagnosis can be delayed.

Patients commonly present with recurrent urinary tract infections, hematuria and abacterial leukocyturia, nephrolithiasis, polyuria/polydipsia with nycturia from infanthood [50,51,52,59,60,61,62] due to impaired urinary concentrating ability [54]. Hypocitraturia is frequently mentioned [45,52,54,55,61,63,64,65,66,67] but urinary acidification capacity has seldom been assessed. A few reports mention distal defect of urinary acidification [45,54,59]: incomplete distal renal tubular acidosis may affect up to 80% of patients [45,66] but it remains unclear whether it is directly caused by CLDN16/19 mutation or by nephrocalcinosis. Hypocitraturia may also be linked to renal failure.

Parathyroid hormone levels seems to be higher than in control patients with chronic renal failure of other origin [49], which may be due to hypomagnesemia-related secondary hyperparathyroidism.

Patients with *CLDN19* mutations frequently display severe ocular abnormalities (myopia, pigmentary retinitis, macular coloboma, strabismus, astigmatism, nystagmus, macular scars, macular degeneration, anisocoria, retinochoroiditis) [50,51,57,58,68], contrasting with milder and rarer ocular abnormalities reported with *CLDN16* mutations (strabismus, myopia, astigmatism, hypermetropia) [45]. CLDN19 is expressed in human fetal retinal pigment epithelium; claudin 19 may be involved in the development and the function of retinal pigment epithelium and retinal neurogenesis [69,70].

Cldn16 and Cldn19 are also expressed in ameloblast tight junction and loss-of-function mutations of *CLDN16* and *CLDN19* genes are associated with amelogenesis imperfecta [71,72]. The lack of Cldn16 strongly impairs tight junction organization in secretory stage ameloblasts [71].

An ophthalmological and a dental evaluation are required.

Muscular-exercise intolerance with pain, weakness and electromyographical alterations [67] has been reported with *CLDN19* mutation but whether this finding is specific of *CLDN19* patients’ needs to be confirmed: hypomagnesemia can lead to non-specific symptoms (muscle weakness and tetany [62]) but Cldn19 is also expressed in murine Schwann cells [73]. Cldn19-deficient mice exhibit behavioral abnormalities that could be due to peripheral neuropathy [73]. Accurate assessment of neuromuscular status in *CLDN16* patients has to be performed in order not to miss subtle abnormalities.

Bone disease [74], bone deformities [62], failure to thrive [50,51,52,62,75] and rickets [45,52,62,76] are reported but their exact pathophysiological mechanisms need to be elucidated.

Histories of nephrolithiasis, nephrocalcinosis, and/or hypercalciuria can be observed in heterozygous family members not affected by FHHNC [45,58,66,67,76] however other causes of these affections are not documented in cases reported.

### 4.2. Prognosis and Treatment

The prognosis of FHHNC is poor as it leads frequently to renal failure in childhood. All patients are affected by nephrocalcinosis but the severity of renal failure is variable.

In order to establish a genotype-phenotype correlation, Konrad et al. studied the function of CLDN16 mutants overexpressed in vitro and classified mutants according to a partial or a total loss of function: the age at onset of symptoms was younger and the decline of GFR was faster in patients bearing *CLDN16* mutations causing a complete loss of function in both alleles [49].

However, there is a clinical heterogeneity of disease severity in families even among patients bearing the same *CLDN16* [45,62,77,78] and *CLDN 19* mutation [58].

Patients with *CLDN19* mutations progress to chronic kidney disease (defined as an eGFR < 60 mL/min/1.73 m^2^) earlier than patients with *CLDN16* mutations and have poorer renal survival [50].

There is no specific therapy for this syndrome. Patients can be treated by thiazide diuretics in order to reduce urinary Ca^2+^ excretion, potassium citrate (as citrate is a crystallization inhibitor) and Mg^2+^ supplements. Additionally, high fluid intake and salt restriction are advised. The effectiveness of these treatments on the course of nephrocalcinosis and on the decline of GFR in FHHNC is unknown. Hydrochlorothiazide has been shown to be effective in correcting hypercalciuria due to *CLDN16* mutations in some [52,79] but not all patients [50,51,52]. Mg^2+^ supplementation might also be ineffective to correct hypomagnesemia [50,51,52]. Hydrochlorothiazide could aggravate hypomagnesemia. Treatment by amiloride can be discussed as potassium-sparing diuretics may have Mg^2+^-sparing properties [80].

Inhibitors of endocytosis may provide novel therapeutic strategies [81,82]. Blocking clathrin-mediated endocytosis increases surface expression of some CLDN16 mutants in vitro. Primaquine, an antimalarial agent increases also cell surface localization of a CLDN16 mutant in vitro [83].

The only treatment of end-stage renal disease is renal replacement therapy. As the primary defect resides in the kidney, there is no recurrence of FHHNC after kidney transplant.

### 4.3. Variants/Pathogenesis

To date, sixty-nine *CLDN16* disease-causing variants have been described including missense/nonsense variants (53), splice site variants (5), small deletions (5), small insertions (2), small indels (2), gross deletions (1), complex mutation (1) (Table 4).

*CLDN16* mutations are located in the two ECSs but also affect the TMDs and the cytoplasmic regions. No link between the affected domain and the phenotype has been described.

The most frequent *CLDN16* disease-causing variant, c.453G>T (p.L151F) is found in almost 50% of the German and Eastern European patients described so far [45,52,100].

Twenty-two *CLDN19* disease-causing variants have been described so far including missense/nonsense variants (19), small deletions (2), gross deletions (1) (Table 5).

Most patients from Spain or southwestern France with *CLDN19* mutation carry the CLDN19 p.(G20D) mutant that may reveal a founder effect [50,51,57,103].

**Figure 2:**
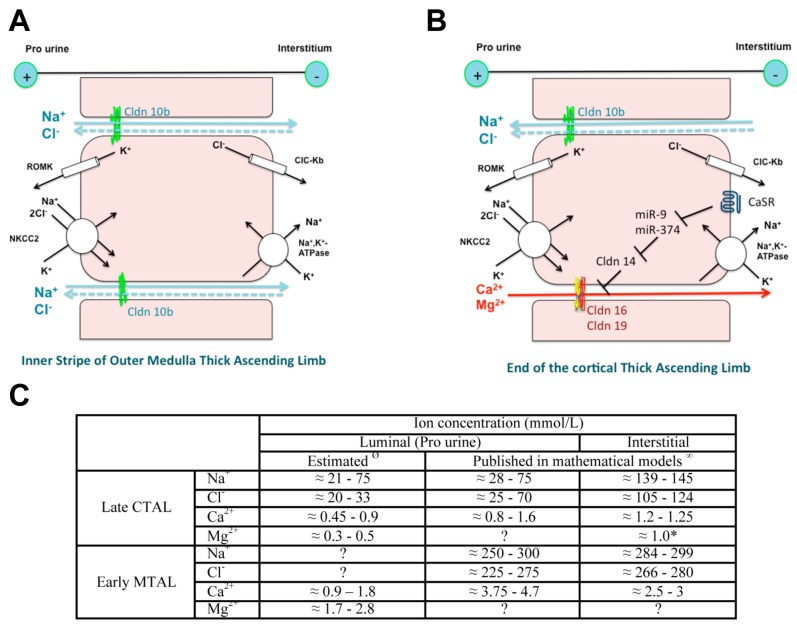
Model of ion transport in the inner stripe of outer medulla (IS-) (**A**) and in the cortical thick ascending limb of the loop of Henle (C-TAL) (**B**) and estimated ion concentration (**C**). The original works used to build the model have been published in References [108,109,110]. NaCl is reabsorbed via the apical cotransporter NKCC2. Most of the potassium that enters the cell recycles back to the lumen via the potassium channel ROMK, thereby hyperpolarizing the apical membrane, while most of the chloride leaves the cell across the basolateral chloride channel CLCKB, resulting in a depolarization of the membrane. Sodium exits the cell via the Na^+^,K^+^-ATPase at the basolateral membrane. The difference in voltage of the two membranes accounts for the lumen positive transepithelial potential difference, the driving force for the paracellular diffusion of divalent cations in the C-TAL and of sodium in the IS-TAL. Claudin (Cldn)16 and Cldn19 may confer a paracellular permeability and selectivity to cations. Cldn14 may interact with Cldn16 and inhibit the Cldn16/Cldn19 complex. It is suggested that Cldn10b drives paracellular NaCl back flux in cortical TAL, adding to the lumen-positive voltage as the paracellular pathway is more permeable to sodium than to chloride. High Ca^2+^ diet and allosteric agonists of calcium-sensing receptor (CaSR) may trigger the expression of Cldn14 via the inhibition of the transcription of two microRNAs miR-9 and miR-374 suppressing *Cldn14* gene expression. ^Ø^ Direct measurement of luminal ion concentration in the TAL is not possible because the segment is inaccessible to micropuncture. Luminal concentrations of Na^+^, chloride (Cl^−^), Mg^2+^ and Ca^2+^ in the early distal convoluted tubule, the segment just downstream the late cortical thick ascending limb (CTAL) have been measured during micropuncture experiments in rodents [111,112,113,114,115,116,117,118,119]. In most studies, concentrations are expressed as a ratio between tubular fluid and plasma ultrafilterable ion concentration. Plasma NaCl is freely filtered, whereas around 60% of Ca and 80% of Mg are ultrafilterable. Luminal ion concentrations in the early medullary thick ascending limb (MTAL) can be estimated based on a Ca^2+^ and Mg^2+^ reabsorption equaling 20–25% and 60–70% of filtered load, respectively and the lack of substantial water reabsorption in the TAL. ^∞^ Values of ion concentrations in the cortical interstitial fluid are similar to those in plasma, due to the dense capillary network and high blood flow in the cortex. Those concentrations have been reported in mathematical models except for magnesium (*). Estimates of the luminal and interstitial concentrations of Na^+^, Cl^−^ and Ca^2+^ in the early medullary thick ascending limbs have been published in mathematical models [120,121,122,123,124,125,126].

Cldn16 and Cldn19 are expressed at the tight junction of the C-TAL of Henle’s loop in rodents and play a key role in the paracellular transport of Ca^2+^ and Mg^2+^. There, 25% of filtered Ca^2+^ and 70% of filtered Mg^2+^ are reabsorbed. The selective transport in the TAL of Ca^2+^ and of Mg^2+^ depends: (1) on a selective paracellular permeability to Ca^2+^ and Mg^2+^ and (2) on a driving force, the lumen-positive transepithelial voltage generated by the active transcellular transport of NaCl and at the end of the C-TAL by NaCl paracellular back flux as the epithelium is more permeable to Na^+^ than to chloride [107] (Figure 2).

**Figure 3:**
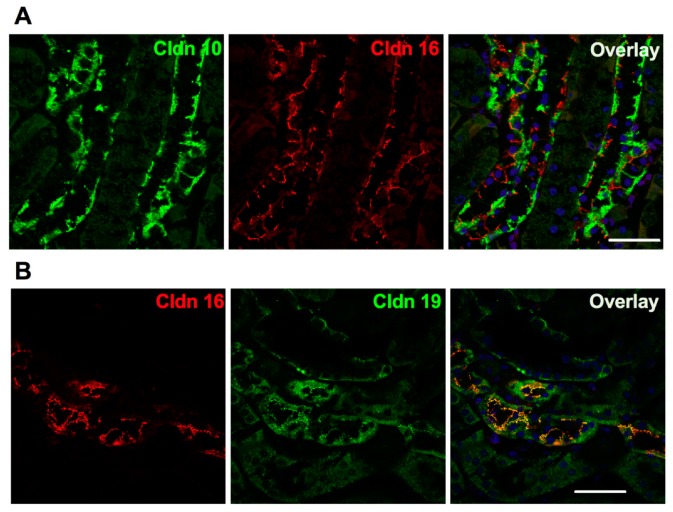
Expression of claudin 10, claudin 16 and claudin 19 in the murine cortical thick ascending limb in immunofluorescence. (**A**) Pattern of expression of claudin 10 and claudin 16 in the mouse C-TAL. Either claudin 10 (Cldn 10, in green) or claudin 16 (Cldn 16, in red) are expressed at tight junction. (**B**) Pattern of expression of claudin 16 and claudin 19 in the mouse C-TAL. Claudin 16 (Cldn 16, in red) and claudin 19 (Cldn 19, in green) are colocalized at tight junction. Bar = 20 µm.

The selective paracellular permeability to Ca^2+^ and Mg^2+^ is most likely conferred by the expression of Cldn16 and 19. In the TAL, Cldn3, 10b, 16 and 19 are expressed under basal conditions [127]. However TAL tight junctions show a mosaic expression of either Cldn10b or Cldn3/Cldn16/Cldn19 in the cortex and in the outer stripe of outer medulla (OS) in mice and rat [127] (Figure 3). Cldn16 is virtually absent from the inner stripe of outer medulla (IS) TAL whereas Cldn10b is highly expressed. Cldn19 is expressed in tight junction and detected intracellularly in OS- and C-TAL whereas it is only detected intracellularly in IS-TAL [127,128]. The Cldn16 proportion of total tight junction length in C- and OS-TAL ranges from 37 to 97% [127]. The permeability ratio P_Mg_/P_Na_ is higher in C- and OS-TAL than in IS-TAL [127].

In patients, bi allelic mutations of *CLDN16* cause a selective defect in paracellular Mg^2+^ and Ca^2+^ reabsorption in the TAL, with intact NaCl reabsorption demonstrated by the normal response to furosemide infusion [54]. However the exact role of claudins 16 and 19 remain a matter of debate. In vitro permeability studies assessing the function of claudin 16 and/or claudin 19 expressed in cell lines have yielded contradicting results. Two hypotheses are been put forward. The first one is that claudin16/claudin19 might function as a divalent cation selective paracellular pore. The second is that claudin16/claudin19 increase P_Na_/P_Cl_, a prerequisite for the diffusion potential in the C-TAL.

Some investigators found that claudin 16 increases transepithelial transport of Ca^2+^ [129] and of Mg^2+^ [83,130,131], when heterogously expressed in epithelial cells (Table 6). Others found an effect of CLDN16 only on Mg^2+^ (not Ca^2+^) permeability [48]. Still others found that CLDN16, when expressed in distinct cell lines, elicits only a small increase in transepithelial Mg^2+^ permeability but a greater increase in Na^+^ permeability [46,47].

The mechanism by which claudin 19 affects Mg^2+^ and Ca^2+^ reabsorption is also unclear according to in vitro heterologous expression studies [47,134] (Table 7).

CLDN 16 and CLDN 19 can interact with each other [47], forming a cis heterodimer at the cell membrane in vitro [135]. Their co expression increases cation selectivity of the tight junction [47]: the effects of CLDN16 and of CLDN19, when co-expressed in LLC-PK1 cells, are synergistic on the P_Na_/P_Cl_ permeability ratio [47]: CLDN19 decreases the permeability to chloride whereas CLDN16 increases the permeability to Na^+^ (Table 5 and Table 6).

The functional consequences of some human *CLDN16* mutations has also been studied by heterologous expression in cells in vitro. Disease-causing mutations can alter intracellular trafficking of claudin 16 or disturb claudin16-claudin 19 interaction or result in a loss of paracellular transport despite its expression at tight junction. Some mutations retain residual function whereas other result in a complete loss of function [46,47,49,81,82,85,96,131]. Similar results have been found with claudin 19 mutants [47,57].

Different mouse models have been engineered to better understand the pathophysiology of FHHNC. Cldn 16 knock-out (KO) model has been engineered a few years ago [136]. Cldn 16 KO mice have hypomagnesemia and hypercalciuria [136]. Lack of Cldn16 decreases P_Ca_/P_Na_ and P_Mg_/P_Na_ in isolated, perfused CTAL whereas P_Na_/P_Cl_ is unaltered [43]. Cldn 16 knockdown (KD) and Cldn 19 KD mice models have been generated by RNA interference technology. Both have hypomagnesemia and significantly increased urinary excretion of Ca^2+^ without significant renal insufficiency [137,138,139]. NaCl absorption may be impaired as Cldn 16 KD mice have low blood pressure and elevated plasma aldosterone concentration. In isolated, perfused TAL from Cldn 16 KD, P_Na_/P_Cl_ is decreased and P_Na_/P_Mg_ is unaffected [139]. Hou et al. conclude that Cldn16 is a non-specific cation channel [139]. The hypothesis is that the mutated Cldn16 protein has an indirect effect on Mg^2+^ reabsorption by decreasing P_Na_/P_Cl_, thereby reducing the diffusion potential/the transepithelial voltage and driving force for Mg^2+^ reabsorption [138,139]. These results are seemingly in conflict with those from Cldn10b cKO mice (see above). However, Cldn10b disruption also lowers P_Na_/P_Cl_ but affects IS-, OS- and C-TAL. In this model, the high transepithelial voltage may elevate passive paracellular Mg^2+^ and Ca^2+^ reabsorption in the C-TAL. Moreover, the mosaic pattern is abrogated and Cldn16 and Cldn19 are expressed in all tight junction in C- and OS- TAL and expression of Cldn 16 expands to IS- TAL, which likely increases the permeability to Mg^2+^ and Ca^2+^ [43,127].

Of note, in mice models (Cldn10 KO, Cldn16 KO, Cldn10/Cldn16 double KO and Cldn 16 KD), bi-ionic diffusion potential may have been measured in the presence of high peritubular MgCl_2_ or CaCl_2_ concentration that activate the basolateral calcium-sensing receptor and make the interpretation of the data more complex [39,43,139].

Renal abnormalities and electrolyte imbalances have not been investigated in *Cldn19* KO mice [73].

Because of the described interaction between Cldn16 and Cldn19, one would have expected a loss of expression of Cldn19 in Cldn16 KO TAL. Surprisingly, the expression of Cldn19 is unaffected in Cldn16 KO mice. The mosaic pattern of expression of either Cldn10b or Cldn3/Cldn19 persisted [127]. In contrast, the majority of Cldn19 tight junctions immunostaining is lost in Cldn16 KD TAL and Cldn16 staining disappeared from tight junctions in Cldn19 KD TAL [137]. Whether the interaction of Cldn16 and Cldn19 is required for their assembly into tight junction is not clear in murine models. Unfortunately, no staining of renal biopsy from FHHNC patient has been performed to our best knowledge.

Renal tissue examination showed Ca^2+^ deposits along the basement membrane of medullary tubules of Cldn16 KD [139] but not of Cldn16 KO mice. None of the models faithfully recapitulate the human disease, they are not complicated by renal failure [136,139]. The pathogenesis of parenchymal deposition of Ca^2+^-containing crystal in the kidney (while Ca^2+^ is less reabsorbed) and of progressive renal failure in FHHNC remains unclear.

## 5. Claudin 14

The human *CLDN14* gene, located on chromosome 21q22.3, contains 1 translated exon, encoding CLDN14, a protein of 239 amino acids [32].

Sixteen *CLDN14* disease-causing variants have been described so far including missense/nonsense variants (12), regulatory substitutions (1) small deletions (2), small indels (1) (13 variants class «DM», 2 variants class «DM?», 1 variant class «Disease-associated polymorphisms with supporting functional evidence DFP») [32].

Mutations in *CLDN14* cause autosomal recessive non-syndromic deafness-29 (DFNB29, OMIM #614035) [140,141], a phenotype reproduced in *Cldn14* KO mice [142]. Heterozygous mutations of *CLDN14* have been described in neonates with vein of Galen malformation [143].

No rare variant of the *CLDN14* gene has been described in Humans with abnormal renal ion handling. However, claudin 14 may have a role in renal Ca^2+^ and Mg^2+^ handling.

Claudin 14 may interact with claudin 16 in TAL and decrease the cation selectivity of the claudin 16-claudin 19 heteromeric complex [144].

The expression of Cldn14 is regulated by changes in dietary Ca^2+^ [144,145]. Cldn14 is either not detected [144] or detected in few murine OS- and C-TAL with distinct location to the tight junction under control conditions; on a high Ca^2+^-containing diet, Cldn14 is highly expressed at murine tight junction of OS- and C-TAL but not in IS-TAL [128]. The expression of Cldn14 is also regulated by chronic changes in dietary Mg^2+^ content in mice and rat [146].

High Ca^2+^ diet and allosteric agonists of calcium-sensing receptor CaSR may trigger the expression of Cldn14 via the inhibition of the transcription of two microRNAs miR-9 and miR-374 suppressing *Cldn14* gene expression [144,145,147].

*Cldn14* KO mice have a higher plasma Mg^2+^ concentration with a lower fractional excretion rate of Mg^2+^ and of Ca^2+^ under high Ca^2+^ dietary condition [144]. On the other hand, overexpression of Cldn14 in TAL generates a phenotype with a lower plasma Mg^2+^ concentration and a higher fractional excretion rate of Mg^2+^ and of Ca^2+^ [147].

When overexpressed in cell culture models, Cldn 14 decreases cation permeability and Ca^2+^ flux [142,145] (Table 8).

Some single-nucleotide polymorphisms (SNPs) of *CLDN*14 are associated with 24 h urinary Ca^2+^ excretion and/or kidney stones [148,149,150,151,152,153]. In silico analysis and in vitro studies of the SNP rs78250838:C> T suggest that it may introduce a novel insulinoma-associated 1 (INSM1) transcription factor binding site, enhancing CLDN14 mRNA and protein expression [151]. Some of these SNPs are also associated with bone mineral density in women [148], serum total CO_2_ [148], Mg^2+^ [150], potassium [150] and PTH [148,150]. One the other hand, Corre et al. identified an SNP (rs172639) in a noncoding intergenic region that was associated with the urinary Mg^2+^ over Ca^2+^ concentration ratio in spot urine [146]. This SNP is part of a large linkage disequilibrium block spanning the 3’ *CLDN14* gene region that contains two microRNA (miR-374 and miR-9) binding sites [144,146].

## 6. Conclusions

Our knowledge regarding claudins in the mammalian kidney and their critical role in ion homeostasis has considerably expanded within the past two decades. This has largely been driven by the need to understand the clinical consequences and provide care to patients affected by rare syndromes caused by genetic mutations of some claudins. The severity of FHHNC and HELIX syndromes underscores the importance of paracellular transport in ion homeostasis. Despite the progresses made in the past years, many questions are left unanswered. Out of the many claudins expressed along the renal tubule and collecting duct, only three have been unequivocally traced to rare renal clinical syndromes in humans. It is quite possible that as yet unidentified syndromes will be recognized as been caused by rare variants of other claudins in the coming years. Besides, a lot has to be understood regarding the physiology of claudins. For example, we do not know which factors determine which claudin(s) is/are expressed at a given tight junction. This is particularly important in the C-TAL where one given cell can make tight junctions with its neighboring cells that differ in claudin composition. Although we know that the properties of tight junctions and hence that of claudins, are tightly regulated [154], we ignored most of the hormones and mechanisms that are involved in the short- and long-term control of claudin function and expression. Finally, providing a specific and effective treatment to patients with claudin-related rare diseases requires that we are able to target mutant claudin to the right tight junction and/or to correct the function of the mutant. The possibility to provide such treatment seems to be far away and will require substantial efforts. However, given the severity of claudin-related rare disease, it is worth the effort.

## Figures and Tables

**Table 1:** Similarities and differences among phenotype in patients with HELIX syndrome.

Reference	Bongers [34]	Klar [35]	Hadj-Rabia [28]	Meyers [36]	Overall (%)
Area of Origin	Europe	Pakistan	North Africa, Pakistan	South America	
Consanguinity	No	Yes	Yes	Yes	
Hypohidrosis	N.D.	13/13	6/6	1/1	20/20 (100%)
Electrolyte imbalance	2/2	6/7	6/6	1/1	15/16 (94%)
Hypolacrimia	N.D.	13/13	6/6	1/1	20/20 (100%)
Ichthyosis	N.D.	N.D.	6/6	0/1	6/7 (86%)
Xerostomia	N.D.	13/13	6/6	1/1	20/20 (100%)
Plasma abnormalities					
Hypokalemia	2/2	0/7	3/6	1/1	6/16 (38%)
Hypermagnesemia	1/2	6/7	6/6	1/1	14/16 (88%)
eGFR < 60 mL/min/1.73 m^2^	1/2	0/3	1/6	1/1	3/12 (25%)
Secondary hyperaldosteronism	N.D.	N.D.	6/6	Hyperaldosteronism without hyperreninism	
Nephrolithiasis	0/2	4/13	0/6	0/1	4/22 (18%)

N.D.: not determined.

**Table 2:** *CLDN10* disease-causing variants [32].

	Missense/Nonsense Mutations					
Ref.	Nucleotide Change	Amino Acid Change	Protein Change	Variant Class	Exon	Domain
[28]	c.2T>C	Met1Thr	p.M1?	DM	1b	Helical
[35]	c.144C>G	Asn48Lys	p.N48K	DM	1b	ECS1
[34]	c.217G>A	Asp73Asn	p.D73N	DM?	1b	ECS1
[36]	c.238A>G	Arg80Gly	p.R80G	DM	2	ECS1
[28]	c.386C>T	Ser131Leu	p.S131L	DM	3	Helical
[34]	c.446C>G	Pro149Arg	p.P149R	DM?	3	ECS2
	**Splicing Mutations**					
[34]	c.465–1G>A		p.E157_T192del	DM?	4	Helical

Variant class is described according the Human Gene Mutation Database [37] DM: Disease-causing mutations; DM?: probable/possible pathological mutation; ECS1: first extracellular segment; ECS2: second extracellular segment.

**Table 3:** Function of claudin 10b according to heterologous expression studies in cell lines.

Claudin	Cell Line	Transfection	TER	P_Na_/P_Cl_	P_Na_	P_Cl_	P_Mg_/P_Cl_	P_Ca_/P_Cl_	Ref.
Mouse Cldn10b	MDCK II	Stable	NS	NS	NS	NS			[33]
Mouse Cldn10b	LLC-PK1	Stable	↘	NS	↗	NS			[33]
Mouse Cldn10b	MDCK-C7	Stable	↘	↗			↗	↗	[38]
Human CLDN10b	MDCK-C7	Stable	↘	↗			↗	↗	[38]
Human CLDN10b	MDCK-C7	Stable	↘						[40]
Mouse Cldn10a	MCDK II	Stable	NS	↘	↘	↗			[33]
Mouse Cldn10a	LLC-PK1	Stable	↘	NS	NS	↗			[33]
Mouse Cldn10a	MDCK II	Stable	NS	↘					[38]
Mouse Cldn10a	MDCK-C7	Stable	NS	NS			NS	NS	[38]
Human CLDN10a	MDCK-C7	Stable	↘	NS			NS	NS	[38]
Human CLDN10a	MDCK-C7	Stable	NS						[40]

In electrophysiological studies, relative epithelial permeabilities (e.g., P_Na_/P_Cl_) are calculated using the Goldman-Hodgkin-Katz equation and the diffusion potential caused by the application of distinct solutions at the apical and basolateral compartment. Absolute permeabilities can be calculated if the transepithelial conductance is known using the Kimizuka-Koketsu equation [41]. The effects of claudin 10a have been included, for comparison with those of claudin 10b. TER: trans epithelial resistance; P_x_: permeability to ion X; NS: not significantly different from control.

**Table 4:** *CLDN16* disease-causing variants [32].

	Missense/Nonsense Mutations					
Ref.	Nucleotide Change	Amino Acid Change	Protein Change	Variant Class	Exon	Domain
[84]	c.114C>A	Cys38Term	p.C38 *	DM ^a^	1	N term
[78]	c.211A>G	Met71Val	p.M71V	DM	1	N term
[26]	c.212T>G	Met71Arg	p.M71R	DM	1	N term
[49]	c.212T>C	Met71Thr	p.M71T	DM	1	N term
[50]	c.239G>A	Cys80Tyr	p.C80Y	DM	1	TM1
[49]	c.263G>A	Gly88Glu	p.G88E	DM	1	TM1
[85]	c.290A>G	Asp97Gly	p.D97G	DM	1	ECS1
[53]	c.295T>G	Trp99Gly	p.W99G	DM	1	ECS1
[49,52]	c.330C>G	Ser110Arg ^b^	p.S110R	DM	2	ECS1
[49,86]	c.341G>A	Arg114Gln	p.R114Q	DM	2	ECS1
[60]	c.340C>T	Arg114Term	p.R114 *	DM	2	ECS1
[56]	c.346C>G	Leu116Val	p.L116V	DM	2	ECS1
[45,66]	c.350G>A	Trp117Term	p.W117 *	DM	2	ECS1
[87]	c.354G>A	Trp118Term	p.W118 *	DM	2	ECS1
[52,88]	c.358T>C	Cys120Arg	p.C120R	DM	2	ECS1
[49]	c.385C>T	Arg129Cys	p.R129C	DM	2	ECS1
[50,54,64,71,86]	c.416C>T	Ala139Val	p.A139V	DM	2	ECS1
[45,49,66,86]	c.421C>G	His141Asp	p.H141D	DM	2	ECS1
[45,46,47,52,66,86]	c.434T>C	Leu145Pro	p.L145P	DM	3	ECS1
[86,89]	c.446G>A	Arg149Gln	p.R149Q	DM	3	ECS1
[45,46,86]	c.446G>T	Arg149Leu	p.R149L	DM	3	ECS1
[26,50]	c.445C>T	Arg149Term	p.R149 *	DM	3	ECS1
[45,46,47,49,52,66,86,88]	c.453G>T	Leu151Phe	p.L151F	DM	3	ECS1or TM2?
[45,49,66,86]	c.452T>G	Leu151Trp	p.L151W	DM	3	ECS1or TM2?
[50,54,71,86]	c.485G>T	Gly162Val	p.G162V	DM	3	TM2
[26,46]	c.500T>C	Leu167Pro	p.L167P	DM	3	TM2
[90]	c.539C>T	Pro180Leu	p.P180L	DM	3	ICL
[50,87,91]	c.547A>G	Lys183Glu	p.K183E	DM	3	ICL
[26,46,47,86]	c.571G>A	Gly191Arg	p.G191R	DM	3	TM3
[65]	c.592G>C	Gly198Arg	p.G198R	DM	3	TM3
[45,86]	c.593G>C	Gly198Ala	p.G198A	DM	4	TM3
[26,46,66,86]	c.593G>A	Gly198Asp	p.G198D	DM	4	TM3
[63]	c.602G>A	Gly201Glu	p.G201E	DM	4	TM3
[92]	c.620G>A	Trp207Term	p.W207 *	DM	4	ECS2
[45,46,47,49]	c.625G>A	Ala209Thr	p.A209T	DM	4	ECS2
[49,59,89]	c.646C>T	Arg216Cys	p.R216C	DM	4	ECS2
[55]	c.647G>A	Arg216His	p.R216H	DM	4	ECS2
[49,93]	c.679G>C	Gly227Arg	p.G227R	DM	4	ECS2
[26,46,47,94]	c.695T>G	Phe232Cys	p.F232C	DM	4	ECS2
[50]	c.697G>C	Gly233Arg	p.G233R	DM	4	ECS2
[26,46]	c.698G>A	Gly233Asp	p.G233D	DM	4	ECS2
[61]	c.697G>T	Gly233Cys	p.G233C	DM	4	ECS2
[52]	c.702G>T	Trp234Cys	p.W234C	DM	4	ECS2
[26]	c.704C>T	Ser235Phe	p.S235F	DM	4	ECS2
[45,46]	c.703T>C	Ser235Pro	p.S235P	DM	4	ECS2
[77]	c.704C>A	Ser235Tyr	p.S235Y	DM	4	ECS2
[49,95]	c.710G>A	Trp237Term	p.W237 *	DM	4	ECS2
[26,45,46,52,66,71,87]	c.715G>A	Gly239Arg	p.G239R	DM	4	ECL2 or TM4?
[52]	c.734G>A	Gly245Asp	p.G245D	DM	4	TM4
[82]	c.823A>T ^c^	Lys275Term	p.K275 *	DM	5	C term
[81]	c.831T>G ^d^	Tyr277Term	p.Y277 *	DM	5	C term
[52]	c.864C>G	Tyr288Term	p.Y288 *	DM	5	C term
[96]	c.908C>G ^e^	Thr303Arg	p.T303R	DM	5	C term
	**Splicing Mutations**					
**Ref.**	**Nucleotide Change**	**Splicing Mutation**		**Variant Class**		
[45]	c.325-5T>G	IVS1 as T-G -5		DM		
[60]	c.427+5G>A	IVS2 ds G-A +5		DM		
[26]	c.593-2A>G	IVS3 as A-G -2		DM		
[49,59]	c.784+1G>T	IVS4 ds G-T +1		DM		
[45]	c.785-14T>G	IVS4 as T-G -14		DM		
	**Small Deletions**					
	**Nucleotide Change**	**Protein Change**		**Variant Class**	**Exon**	**Domain**
[97]	c.166delG ^f^	p.(Ala56Leufs*16)		DM?	1	N term
[49]	c.235delG ^g^	p.(Ala79fsX90)			1	TM1
[45]	c.368delA	p.(Asn123Metfs*21)		DM	2	ECS1
[49]	c.408_410delCAT	p.(Ile137del)		DM	2	ECS1
[61]	c.800delG	p.(Arg267Lysfs*7)		DM	5	C term
	**Small Insertions**					
	**Nucleotide Change**	**Protein Change**		**Variant Class**	**Exon**	**Domain**
[74]	c.324+3_324+4insT	Not available		DM	intron 1	
[71]	c.545_548dupTTAA	p.(Lys183Asnfs*2)		DM	3	ICL
	**Small Indels**					
	**Nucleotide Change**	**Protein Change**		**Variant Class**	**Exon**	**Domain**
[45,66]	c.165_166delGGinsC	p.(Arg55Serfs*17)		R	1	N term
[45,46]	c.646_647delCGinsAC	p.(Arg216Thr)		DM	4	ECS2
	**Gross Deletions**					
	**DNA Level**	**Description**		**Variant Class**	**Exon**	**Domain**
[71,98]	g.DNA	Ex. 2-5		DM	2-5	
	**Complex Mutations**					
		**Description**		**Variant Class**	**Exon**	**Domain**
[62]		c.574_589delins23bp	p.(A192Yfs∗ 25)	DM	3	TM3

Variant class is described according the Human Gene Mutation Database [37] DM: Disease-causing mutations; DM?: probable/possible pathological mutation; «Retired records (R)», a variant that has been removed from HGMD if found to have been erroneously included ab initio or if the variant has been subject to retraction/correction in the literature resulting in the record becoming obsolete, merged or otherwise invalid. Domains are described according to authors and [99]. C Term, COOH terminus; TM, transmembrane domain; ECS1, first extracellular segment; ICL, intracellular loop; ECS2, second extracellular segment; N Term, NH_2_ terminus. ^a^: the «significance» described by Trujillano was “likely pathogenic according to ACMG guidelines.” They categorized patients’ phenotypes according to the Human Phenotype Ontology nomenclature based on the clinical data and preceding workup provided by the referring physician. The phenotype described was: psychosis, seizures, muscle weakness, respiratory failure, reduced dihydropyrimidine dehydrogenase activity, decreased body weight, reduced consciousness/confusion, lower limb muscle weakness; ^b^: reported as p.S110R 329AGC>AGG; ^c^: reported as p.L203*, c.822A>T; ^d^: reported as p.Y207*, c.620T>G; ^e^: reported as p.T233R, c.697C>G; ^f^: reported as c.164delG; ^g^: reported as 236delG, p.A80fsX91,; * indicates that the predicted consequence is a termination codon.

**Table 5:** *CLDN19* disease-causing variants [32].

	Missense/Nonsense Mutations					
Ref.	Nucleotide Change	Amino Acid Change	Protein Change	Variant Class	Exon	Domain
[50]	c.54G>A	Trp18Term	p.W18 *	DM	1	TM1
[47,50,51,57,58,67,70,72,101,102,103]	c.59G>A ^a,b^	Gly20Asp	p.G20D	DM	1	TM1
[50,101,102]	c.83C>T	Pro28Leu	p.P28L	DM	1	TM1
[51]	c.122T>C	Ile41Thr	p.I41T	DM	1	ECS1
[50,67]	c.130G>A	Val44Met	p.V44M	DM	1	ECS1
[47,51,57]	c.169C>G	Gln57Glu	p.Q57E	DM	1	ECS1
[50,72]	c.169C>T	Gln57Term	p.Q57 *	DM	1	ECS1
[51]	c.223G>T	Gly75Cys	p.G75C	DM?	1	ECS1
[51]	c.223G>A	Gly75Ser	p.G75S	DM?	1	ECS1
[68,76] ^c^	c.241C>T	Arg81Trp	p.R81W	DM	2	ECS1 or TM2?
[104]	c.263T>A	Val88Glu	p.V88E	DM	2	TM2
[72]	c.269T>G	Leu90Arg	p.L90R	DM	2	TM2
[47,57]	c.269T>C	Leu90Pro	p.L90P	DM	2	TM2
[51]	c.364G>A	Gly122Arg	p.G122R	DM	2	TM3
[63]	c.388G>T	Gly130Asp	p.G130C	DM	2	TM3
[63,75]	c.389G>A	Gly130Asp	p.G130D	DM	3	TM3
[105]	c.506G>A ^d^	Trp169Term	p.W169 *	DM	4	TM4
[94]	c.535G>A	Gly179Ser	p.G179S	DM	4	TM4
[72]	c.599G>A	Arg200Gln	p.R200Q	DM?	4	C term
	**Small Deletions**					
	**Nucleotide Change**	**Protein Change**		**Variant Class**	**Exon**	**Domain**
[106]	c.140_141delAT	p.(Tyr47 *)		DM	1	ECS1
[50]	c.403_406delACTG	p.(Thr135Leufs*9)		DM	3	TM3
	**Gross Deletions**					
	**DNA Level**	**Description**		**Variant Class**	**Exon**	**Domain**
[50]	g.DNA	Ex. 1-4		DM	1-4	

Variant class is described according the Human Gene Mutation Database [37]. DM: Disease-causing mutations; DM?: probable/possible pathological mutation. Domains are described according to authors and [99]. C Term, COOH terminus; TM, transmembrane domain; ECS1, first extracellular segment; ICL, intracellular loop; ECS2, second extracellular segment; N Term, NH_2_ terminus. ^a^: reported as c.C>T in ref [58]; ^b^: reported as c.69G>A in ref [72]; ^c^: reported as p.Arg81Cys in ref [68]; ^d^: reported as c.697G>A in ref [105]; * indicates that the predicted consequence is a termination codon..

**Table 6:** Function of claudin 16 according to heterologous expression studies in cell lines.

Claudin	Cell Line	Transfection	TER	P_Na_/P_Cl_	P_Na_	P_Cl_	P_Mg_	P_Ca_	Mg^2+^ Flux	Ca^2+^ Flux	Ref.
Rat Cldn16	MDCK	stable	↗							↗ ^*∫◊^	[129]
Rat? Cldn16	MDCK	stable	↗	↘	↘	NS			↗ ^§ø^		[130]
Rat Cldn16	MDCK Tet-OFF	Inducible expression	↗						↗ ^§*^		[132]
Human ∆70 CLDN16	LLC-PK1	Stable?	↘	↗	↗	NS	↗ ^†^				[46]
Human ∆70 CLDN16	MDCK II	Stable?			NS	NS	NS				[46]
Full length Human CLDN16	LLC-PK1	Stable?	↘	↗	↗	NS					[47]
Full length Human CLDN16	MDCK II	Stable?			NS	NS					[47]
Short and long version of human CLDN16	MDCK-C7	Stable				***	↗ ^£^	NS ^∫^			[48]
Long version of human CLDN16	MDCK-C7	Stable			NS		↗ ^∞^	NS ^∞^			[48]
Full-length human CLDN16	MDCK-C7	stable		NS			↗ ^¥^				[131]

Relative epithelial permeabilities (e.g., P_Na_/P_Cl_) are calculated using the Goldman-Hodgkin-Katz equation and the diffusion potential caused by the application of distinct solutions at the apical and basolateral compartment. Absolute permeabilities can be calculated if the transepithelial conductance is known using Kimizuka-Koketsu equation [41]. Measuring ion flux can be performed using either radioactive isotopes or fluorescent compounds or non-radioactive ions. Flux measurement include trans- and paracellular transport [41]. TER: trans epithelial resistance; NS: not significantly different from control; P_x_: permeability to ion X; ^∫^: Transepithelial transport of ^45^Ca^2+^ measurement; ^§^: The transepithelial transport of Mg^2+^ was measured using Xylidyl Blue-I.; ^†^: The permeability of Mg^2+^ across monolayers was determined according to Tang and Goodenough [133]; ^£^: Mg^2+^ flux was measured employing atomic absorption spectrometry; ^∞^: Mg^2+^ and Ca^2+^ permeabilities, calculated from dilution potential/bionic potential measurements; ^¥^: Measurement of unidirectional fluxes from the basolateral to the apical side was performed under short-circuit conditions with MgSO_4_. The atomic absorption of Mg^2+^ was measured in an oxidizing air-acetylene flame at 285.2 nm. P_Mg_ was calculated from resulting fluxes (P_Mg_ = flux/concentration); ∂Permeability PC = Flux/Substrate concentration in cis compartment. This was then corrected for the permeability of blank filters, PB, to obtain the true transepithelial permeability (PT), using the following equation PT = [(1/PC) & (1/PB)]^−1^; ***: Increased basolateral Mg^2+^ concentration induces a short circuit current (may activate a transcellular Cl^−^ current); *: from apical to basal compartment, without affecting transport from basal to apical compartments; ^ø^: from apical to basal compartment; ^◊^: The apical to basolateral flux was competitively inhibited by Mg^2+^.

**Table 7:** Function of claudin 19 and of claudin 16 and claudin 19 co-expression according to heterologous expression studies in cell lines.

Claudin	Cell Line	Transfection	TER	P_Na_/P_Cl_	P_Na_	P_Cl_	P_Mg_	P_Ca_	Ref.
Human CLDN19	LLC-PK1	Stable?	↗	↗	NS	↘	^†^		[47]
Human CLDN19	MDCK II	Stable?			NS	NS			[47]
Mouse Cldn19	MDCK II Tet-Off cells	Stable inducible expression	↗		↘	NS	↘ ^£∂^	↘^∫∂^	[134]
Human CLDN19 + full length human CLDN16	LLC-PK1	Stable?	↘	↗	↗	↘	↘ ^†^		[47]

Relative epithelial permeabilities (e.g., P_Na_/P_Cl_) are calculated using the Goldman-Hodgkin-Katz equation and the diffusion potential caused by the application of distinct solutions at the apical and basolateral compartment. Absolute permeabilities can be calculated if the transepithelial conductance is known using Kimizuka-Koketsu equation [41]. TER: trans epithelial resistance; NS: not significantly different from control; P_x_: permeability to ion X; ^∫^: Transepithelial transport of ^45^Ca^2+^ measurement; ^†^: The permeability of Mg^2+^ across monolayers was determined according to Tang and Goodenough [133]; ^£^: Mg^2+^ flux was measured employing atomic absorption spectrometry; ^∂^: Permeability PC = Flux/Substrate concentration in cis compartment. This was then corrected for the permeability of blank filters, PB, to obtain the true transepithelial permeability (PT), using the following equation PT = [(1/PC) & (1/PB)]^−1^.

**Table 8:** Function of claudin 14 according to heterologous expression studies in cell lines.

Claudin	Cell Line	Transfection	TER	P_Na_/P_Cl_	P_Na_	P_Cl_	P_Ca_	Ca^2+^ Flux	Ref.
Human CLDN14	MDCK II Tet-Off cells	Stable inducible expression	↗	↘	↘				[142]
Mouse Cldn14	OK	Stable	↗	↘	↘	NS		↘∫ ^ø^	[145]
Mouse Cldn14	MDCK II Tet-Off cells	Stable inducible expression	↗	↘	↘	NS	↘^∫^		[145]

Relative epithelial permeabilities (e.g., P_Na_/P_Cl_) are calculated using the Goldman-Hodgkin-Katz equation and the diffusion potential caused by the application of distinct solutions at the apical and basolateral compartment. Absolute permeabilities can be calculated if the transepithelial conductance is known using Kimizuka-Koketsu equation [41]. Flux measurement include trans- and paracellular transport [41]. TER: trans epithelial resistance; NS: not significantly different from control; P_x_: permeability to ion X; ^∫^: Transepithelial transport of ^45^Ca^2+^ measurement; ^ø^: from apical to basal compartment.

## Abbreviations

CLDN	human claudin protein
CLDN	human claudin gene/mRNA
Cldn	rodent claudin protein
Cldn	rodent claudin gene/mRNA))
